# Real-world Adoption of Smartphone-based Remote Monitoring Using the Confirm Rx™ Insertable Cardiac Monitor

**DOI:** 10.19102/icrm.2021.120806

**Published:** 2021-08-15

**Authors:** Roland R. Tilz, Naushad Shaik, Christopher Piorkowski, Yajing Hu, Allison T. Connolly, Ignacio J. Reyes, Yelena Nabutovsky, Avi Fischer, John Ip

**Affiliations:** ^1^Division of Electrophysiology, Medizinische Klinik II (Kardiologie, Angiologie, Intensivmedizin), Universitäres Herzzentrum Lübeck, Lubeck, Germany; ^2^Cardiovascular Associates, Orlando, FL, USA; ^3^Heart Center, Dresden, Germany; ^4^Abbott, Santa Clara, CA, USA; ^5^Abbott, Sylmar, CA, USA; ^6^Sparrow Cardiovascular Institute, Lansing, MI, USA

**Keywords:** Atrial fibrillation, connectivity, insertable cardiac monitor, remote monitoring, syncope

## Abstract

While previous generations of insertable cardiac monitors (ICMs) required a bedside monitor for remote monitoring (RM), the Confirm Rx™ ICM (Abbott, Chicago, IL, USA) utilizes Bluetooth^®^, Wi-Fi/cellular technology, and a smart device to connect to the RM system. We aimed to characterize compliance, connectivity, and event transmission timing with the Confirm Rx™ ICM RM system. The study cohort included American patients who received the Confirm Rx™ ICM with SharpSense™ technology within three months of release (May–July 2019). Compliance with RM was quantified as the proportion of patients registering the patient app on their smart device and transmitting at least once. Connectivity was measured as the median number of days between consecutive transmissions per patient. Event transmission time was measured from episode detection to availability on the Merlin.net™ RM system (Abbott). Time from transmission until review by a clinician was examined. Values for device connectivity, episode transmission timing, and clinician view times were reported as median [first quartile, third quartile]. Of 5,666 patients who received a Confirm Rx™ ICM, 97% registered their patient app and 92% transmitted data at least once. Among those utilizing RM (aged 66 ± 15 years; 49% female), connectivity occurred every 1.5 [1.2, 2.4] days, or 4.7 times per week. Patient-reported symptoms were transmitted to Merlin.net™ within 2.9 [2.1, 3.8] minutes of event onset and viewed by the clinician within 0.9 [0.4, 3.1] days, while device-detected episodes without symptoms were transmitted within 18.5 [11.2, 36.5] hours and then viewed within 0.8 [0.3, 2.5] days. This real-world study demonstrated excellent patient compliance with the smartphone-based RM paradigm enabled by Confirm Rx™, suggesting the suitability of this technology for future cardiac implantable devices.

## Introduction

Insertable cardiac monitors (ICMs) are effective in the early detection of arrhythmias that are too infrequent to be recorded by traditional 24- to 48-hour Holter monitors,^1^ and their use has been established for clarifying mechanisms responsible for unexplained syncope and palpitations.^2,3^ There is increasing evidence demonstrating the value and effectiveness of ICMs in the detection of atrial fibrillation (AF) after cryptogenic stroke,^4,5^ ablation,^6–10^ and postoperatively.^11^ ICMs are also being used to quantify the arrhythmia burden in patients with known AF,^12^ and early investigations have shown the feasibility of, and economic benefits associated with, ICM use in the management of oral anticoagulation used in AF patients.^13,14^

However, the value of the ICM is dependent on the effective and timely transmission of ICM-detected events to the clinician. Prompt enrollment in and high adherence to remote monitoring (RM) for cardiac implantable electronic devices (CIEDs) have been shown to improve survival and reduce health care utilization.^15–17^ The recent worldwide coronavirus disease 2019 (COVID-19) pandemic has highlighted the importance of effective telemonitoring. While previous generations of ICMs used radiofrequency technology and required a bedside monitor for RM, the Confirm Rx™ (Abbott, Chicago, IL, USA) is a smart device–enabled ICM that utilizes Bluetooth^®^ and Wi-Fi/cellular technology along with a specially developed myMerlin™ smartphone application (app) (Abbott) to connect to the RM system. In this large, retrospective real-world study, we aimed to evaluate patient compliance with the smartphone patient app, connectivity between the patient and clinician, and timeliness of episode transmissions.

## Methods

### Study cohort and patient selection

The study cohort included all American patients implanted with a Confirm Rx™ ICM within the first three months of release of the SharpSense™ technology update (Abbott), from May 1, 2019, to July 31, 2019. SharpSense™ contains new discrimination algorithms to improve the accuracy of event detection and only patients with this technology were studied to reflect the user experience with the latest generation of devices. Patients were included regardless of their indication for cardiac monitoring. Connectivity and transmission times were evaluated in those patients who registered in the Merlin.net™ RM network and had SharpSense™ activated. Datasets from Merlin.net™ were de-identified prior to the analysis and publication, as defined by the Health Insurance Portability and Accountability Act (HIPAA) of 1996 in 45 CFR Section 164.514(b) implementation specifications: requirements for de-identification of protected health information.

### The device

The Confirm Rx™ ICM is a minimally invasive, implantable cardiac monitor. The SharpSense™ technology improves the accuracy of detection by utilizing a bradycardia-undersensing discriminator and implementing pause-undersensing and loss-of-contact discriminators. Automatic detection of an episode, including ventricular tachycardia, bradycardia, AF, or a pre-defined duration of pause, triggers electrocardiogram (EGM) storage within the ICM and sets an alert flag. A patient can also record symptoms and trigger manual EGM storage at any time through the app, which activates the symptom alert. Each alert type can be activated or deactivated by the clinician, as can the determination of priority for a particular type of transmission. For example, in patients implanted with the device for AF monitoring, only AF alerts might be activated and other alerts deactivated.

Patients utilized either their own or an Abbott-provided smartphone for RM. The Bluetooth^®^ technology enables communication between the Confirm Rx™ ICM and the myMerlin™ smartphone app, which then transmits data to the Merlin.net™ patient care network via Wi-Fi or a cellular service. There are several types of transmissions that can occur with this system, including daily connectivity checks, scheduled monthly transmissions, and patient-initiated transmissions. Daily connectivity checks transmit data from the Confirm Rx™ device to Merlin.net™ every day regardless of the presence of any alerts. If an activated alert condition was triggered, that episode was transmitted during the daily check. Scheduled monthly transmissions occur every 31 days and transmit all alerts (activated and deactivated). Patient-initiated and/or symptom-driven transmissions are sent to Merlin.net™ immediately.

### Compliance with remote monitoring and device connectivity

Compliance was quantified as the percent of patients who registered their ICM with the myMerlin™ smartphone app and sent at least one Merlin.net™ transmission during the study period. Device connectivity was defined as the median number of days between consecutive transmissions for each patient. The follow-up period was defined as the number of days between the ICM implant and the last transmission. Device connectivity was calculated in patients with at least two transmissions over the follow-up period. The effects of age and sex on device connectivity using this novel platform were studied.

### Episode transmission and clinician view times

Episode transmission timing was calculated separately for patient-initiated transmissions and routine device-initiated events. For device-initiated events, the timing was analyzed only for alert types that were activated by the physician. The median time from episode detection to transmission to availability within Merlin.net™ is reported overall and separately for different alert types. The effect of age and gender on episode transmission timing was evaluated. Clinician view time was the median number of days from episode availability in Merlin.net™ to clinician review online.

### Statistical analysis

Values for device connectivity, episode transmission timing, and clinician view times were reported as median [first quartile, third quartile] across the study cohort and across each subgroup. Median values were used due to the data having a non-normal distribution.

The effect of patient characteristics, including age and gender, on device connectivity was tested using the Anderson–Gill model, where recurrent events were measured as the server interactions from each patient. Patient identifiers were clustered for the robust estimate of standard errors.

The stability of connectivity over time was evaluated using the Kruskal–Wallis test by rank to test whether there were any differences in the average days between transmissions in the first four months.

Linear mixed-effects models were built to evaluate the effect of age and gender on the episode transmission timing. The patient identifier was treated as the random effect in the models to account for variations in the repeated measurements from each patient.

RStudio version 1.2.5019 (RStudio, Boston, MA, USA) with R version 3.6.1 (R Foundation for Statistical Computing, Vienna, Austria) was used for statistical analysis.

## Results

### Compliance

**Figure 1:**
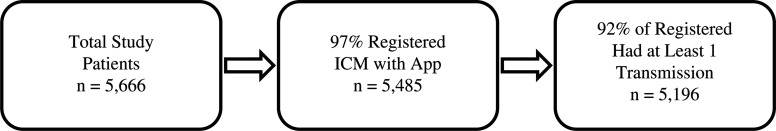
Compliance with RM. A high proportion of patients registered their implantable cardiac monitor with the patient app and then transmitted data to the physician. ICM: insertable cardiac monitor.

Overall, 5,666 patients were evaluated and comprised the study cohort to study compliance with RM. Of these, 5,485 (97%) patients registered the ICM with the myMerlin™ patient app and 5,196 (92%) patients transmitted data at least once during the study period **(Figure 1)**. Neither the percent registered nor the percent with transmission differed by age. Of note, patients aged 85 years or older had the same registration rate as the rest of the cohort.

### Connectivity

Out of the 5,196 patients who met the compliance requirement, 4,605 patients utilized SharpSense™ technology and were further evaluated for connectivity and transmission timing. The median follow-up period was 74 [51, 99] days. The average age was 66 ± 15 years, and of those with sex information available, 49.8% were women. The majority of the study participants were implanted with an ICM for the evaluation of syncope. Patient characteristics, including the reason for monitoring, are presented in **Table 1**.

**Figure 2:**
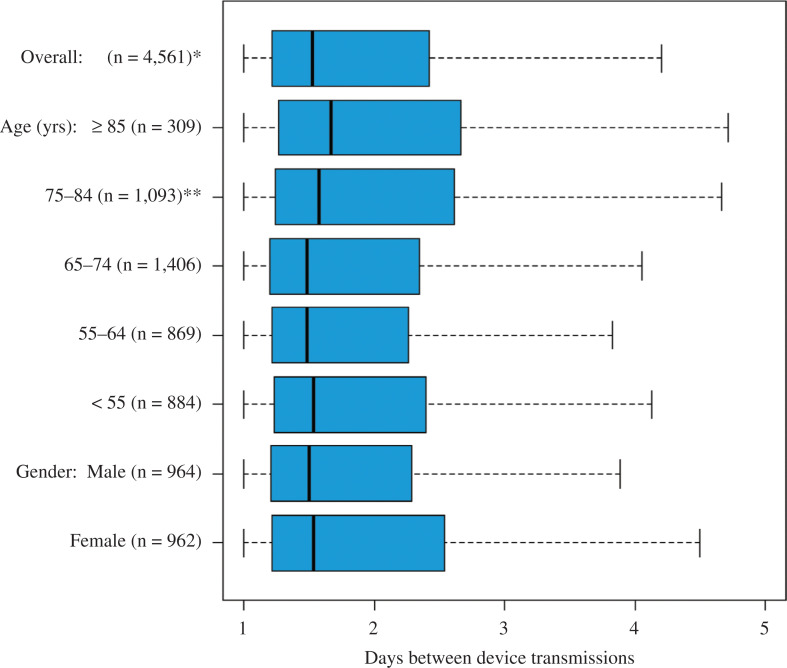
Connectivity—days between daily transmissions. The figure displays boxplots of days between transmissions for various patient groups. Each boxplot shows the median value as a solid line, while the limits of the box represent the 25^th^ and 75^th^ quartiles and the outer edges are the minima and maxima, respectively. For comparison between age groups, patients younger than 55 years of age were used as a reference group, which all other age groups were compared against. *The number of patients only includes those with at least two transmissions. **Indicates a significant difference.

**Figure 3:**
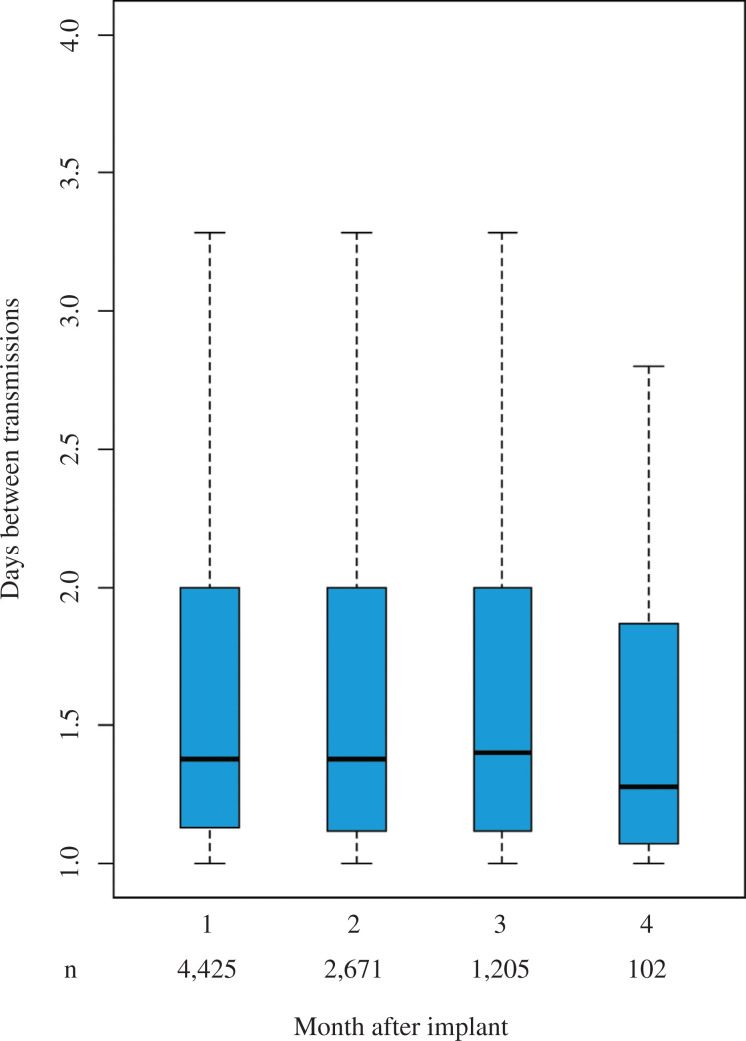
Stability of connectivity over time. The figure displays boxplots of days between transmissions each month after implant. Each boxplot shows the median value as a solid line, while the limits of the box represent the 25^th^ and 75^th^ quartiles and the outer edges are the minima and maxima, respectively. The number of patients available for analysis in each month is displayed below the x-axis.

Transmissions from the ICM to Merlin.net™ occurred every 1.5 [1.2, 2.4] days **(Figure 2)** and remained stable over the first four months after the implant **(Figure 3)** (p = 0.325). There was no difference in transmission frequency between men and women (p = 0.051). Patients younger than 55 years were used as the reference group against which all others were compared. There were no statistically significant differences in connectivity among patients younger than 75 years and no differences between younger patients and those aged older than 85 years. There was a small but statistically significant difference between those aged younger than 55 years and those aged 75 to 85 years, with those aged 75 to 85 years transmitting slightly less frequently, every 1.57 [1.24, 2.62] days, compared to those aged younger than 55 years, who transmitted every 1.53 [1.23, 2.39] days (p = 0.009).

### Transmission timing

**Figure 4:**
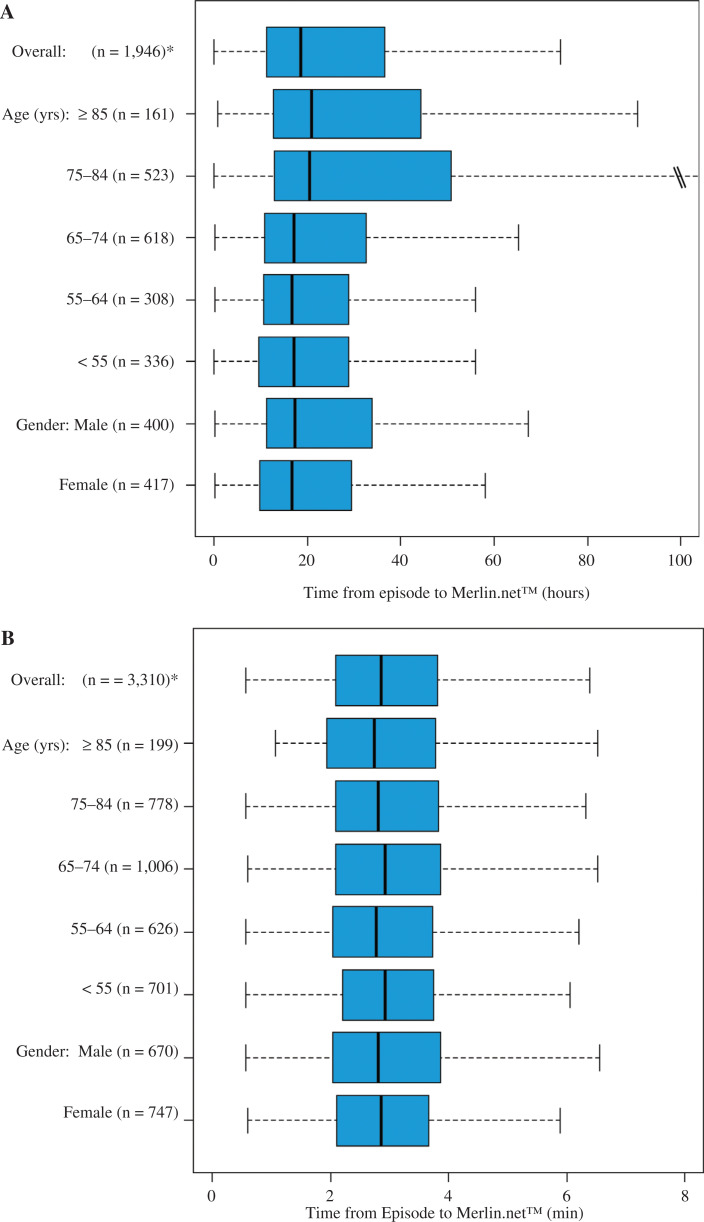
Effect of patient characteristics on the timing of transmissions for (A) device-initiated and (B) patient-initiated transmissions. The figure displays boxplots of time from episode detection to Merlin.net™ availability. Each boxplot shows the median value as a solid line, while the limits of the box represent the 25^th^ and 75^th^ quartiles and the outer edges are the minima and maxima, respectively. *The number of patients only includes those with episodes.

Device-detected episodes were transmitted from the ICM to Merlin.net™ within 18.5 [11.2, 36.5] hours and then viewed by the clinician within 0.8 [0.3, 2.5] days from transmission **(Table 2)**. The timing of transmission did not differ by age (p = 0.361), but there was a statistically significant difference by sex **(Figure 4A)** (p = 0.030). Women transmitted episodes within 16.7 [9.7, 29.3] hours, while men took 17.3 [11.3, 33.8] hours to transmit. All types of device-detected episodes were transmitted within a similar amount of time **(Table 3)**. Patient-initiated transmissions associated with symptoms were transmitted within 2.9 [2.1, 3.8] minutes, with 96% of episodes being transmitted within one hour. Symptom episodes were subsequently viewed by the clinician within 0.9 [0.4, 3.1] days **(Table 2)**. There were no differences in symptom episode transmission time by age or sex **(Figure 4B)** (p = 0.279 for the effect of age; p = 0.259 for the effect of sex).

## Discussion

This large real-world study aimed to evaluate the compliance, connectivity, and timeliness of episode transmission in patients who received the new smartphone-enabled Confirm Rx™ implantable cardiac monitor. We report very high compliance with the patient app, with 97% of patients activating the app and 92% sending at least one transmission. We also observed consistent connectivity between patients and the RM platform, with a median time between transmissions of 1.5 days. Finally, episode transmission occurred quickly—within 2.9 minutes for patient-initiated transmissions and within 18.5 hours for device-detected events. While clinicians took approximately one additional day to view transmissions after they became available, the fact that patients can initiate transmissions of device data when they have symptoms and that these data are available to the treating physicians within a few minutes may create opportunities for real-time patient-centric interventions.

There are numerous publications both from randomized clinical trials and large real-world cohorts showing the clinical and economic benefits of RM in traditional CIEDs, such as pacemakers, defibrillators, and cardiac resynchronization therapy devices.^18^ Therefore, international physician societies have designated RM as a class I indication and believe that RM represents the new standard of care for patients with CIEDs.^19^ Furthermore, the COVID-19 pandemic has highlighted the importance of effective telemonitoring. The RM technology has evolved, progressing from telephone-based follow-ups to web-based monitoring. With the fast pace of technological advancements and advent of new communication protocols, it is important to study the impact of these changes on patient compliance and management. Although the Bluetooth^®^ technology has become widespread in other areas, it is new to CIEDs, and its impact on RM and patient care requires evaluation.

Past investigations of real-world data have demonstrated that enrollment in traditional RM is relatively low.^17,20^ For example, an investigation of a large CIED RM system reported that only 61% of patients with RM-eligible devices were enrolled in RM and 79% of these patients transmitted data.^20^ In contrast, in this analysis, we found that 97% of patients who received the Confirm Rx™ device registered their ICM with the smartphone app and 92% transmitted data at least once. One reason for the difference might be the more demanding system setup requirements for traditional RM, which depend on telecommunications infrastructure, the ability of the patient to set up the device, the need for a separate transmitter that is not always portable, and the commitment required from health care providers to provide support. An alternative explanation for differences between ICM and CIED RM could be the purpose of these devices—pacemakers and defibrillators are implanted to provide therapy, while the main purpose of the ICM is to establish a diagnosis or monitor for occult, often infrequent arrhythmias, many times leading to changes in clinical management strategies. This might provide more motivation for the use of RM with ICMs compared to other CIEDs.

Since the advent of RM, several trials have reported on the efficacy and timeliness of episode transmissions.^21^ Systems that relied on phone lines often suffered from technical shortcomings. For example, the Clinical Evaluation of Remote Notification to Reduce Time to Clinical Decision (CONNECT) study, which evaluated an early-generation RM system, reported that 45% of alerts did not get transmitted to the clinic, mainly because the home monitor was not properly set up.^22^ Even when the alerts were transmitted, the time from the alert to clinical decision-making was 4.6 days on average and only 84% of these alerts were received in a timely manner.^22^ The Implant-based Multiparameter Telemonitoring of Patients with Heart Failure (IN-TIME) trial reported gaps in data transmission when patients were away from home for three or more consecutive days.^23^ On the other hand, the Evolution of Management Strategies of Heart Failure Patients with Implantable Defibrillators (EVOLVO) trial, in which all patients in the RM arm were required to fully set up and learn how to use the system, the median time from the alert to review by the clinician was only 1.4 days.^24^ In the Monitoring Resynchronization Devices and Cardiac Patients (MORE-CARE) randomized controlled trial, the median delay from device-detected events to clinical decisions was 2 [1, 4] days in the RM group and 29 [3, 51] days (p = 0.004) in the control group that had a standard follow-up without alerts.^25^

In our real-world analysis, we observed a relatively short timeline from episode detection to clinician review, specifically, one day for patient-initiated and 1.5 days for device-detected episodes. The more rapid data transmission may reflect the use of Bluetooth^®^ technology, which allows for the transmission of data from anywhere, not just from home where a bedside transmitter is traditionally located. With Confirm Rx™, the only requirements for transmission are carrying a smart device and having access to Wi-Fi or a cellular service.

When considering adoption and use of a new technology that requires patient involvement and the use of smartphone-based technology, the age of the patient may be an important consideration. We found small but statistically significant differences between age groups in the frequency of transmissions. There were no differences between subgroups of patients younger than 75 years or those over 85 years, but the age group of 75- to 85-year-old patients transmitted slightly less frequently (every 1.57 days vs. every 1.53 days for the overall study cohort) than younger patients. Those aged over 85 years also trended toward a slightly lower frequency of transmission, but did not reach significance, likely due to the smaller sample size of this older age group. There were no differences in transmission time by age for device-initiated or patient-initiated episodes. Overall, the elderly and the extremely elderly exhibited strong compliance with smartphone-based RM, almost on par with the rest of the cohort, providing important evidence for smartphone-based medical technology in this growing population. The effect of patient age on the ability to use RM has not been extensively investigated. A large real-world study of remotely monitored CIED patients that conservatively defined RM compliance as transmitting data at least twice in a 14-month period found that patients aged over 80 years were the most compliant, closely followed by those aged 66 to 80 years, with patients below 66 years of age lagging far behind.^20^ Importantly, the system evaluated in that study utilized a bedside monitor, in contrast to the smartphone-enabled system used with Confirm Rx™. A recent behavioral health investigation that specifically evaluated mobile health use among older adults found that a significant portion of older adults already utilize mobile technology, that they are willing to engage with mobile technology for health reasons, and that their overall attitude toward mobile technology is positive.^26^ Therefore, it is not surprising that, in our analysis, older adults performed similar to the overall cohort in terms of connectivity and transmission time.

The Bluetooth^®^ technology is becoming ubiquitous in electronic devices, the use of smartphones is widespread globally, and the vast majority of the world is connected online. Many patient lifestyle and health apps have been deployed globally, ranging from those used to manage activity, diet, sleep, and overall general health to those specialized for monitoring and managing chronic diseases. While it is clear that people are becoming familiar with the concept of using mobile technology to monitor health, researchers struggle to keep up with the innovation and evaluate the quality and utility of these tools. CIEDs and ICMs are in a unique category of devices that have been shown to provide benefit through numerous clinical trials, but their union with the latest available communication technology may provide an opportunity for further improvement.

### Limitations

Limited information about the clinical characteristics of the patients in this study was available. As this was an RM dataset, only age, sex, and the reason for cardiac monitoring were provided. Patient characteristics such as comorbid conditions and socioeconomic factors may impact the efficacy of RM. In addition, this analysis was limited to the American population, so it may not reflect the user experience in other parts of the world.

## Conclusion

Implantable cardiac monitors are increasingly being used for the detection of and monitoring for arrhythmias. An efficient and timely transmission of information from the device to the clinician is imperative for optimal patient outcomes. As medical technologies continue to improve, it is important for the clinical community to examine the effects of these technologies on patient care, patient compliance, and patient connectivity. We found that Confirm Rx™, which utilizes Bluetooth^®^ and Wi-Fi technologies for RM, has a very high rate of patient compliance, excellent connectivity, and fast episode transmission times.

## Figures and Tables

**Table 1:** Patient Characteristics

Variable	Data
Total number of patients	4,605
Follow-up period*	74.0 [51.0, 99.0] days
Age, mean ± standard deviation	66 ± 15 years
< 55 years, n (%)	891 (19%)
55–64 years, n (%)	875 (19%)
65–74 years, n (%)	1,423 (31%)
75–84 years, n (%)	1,105 (24%)
≥ 85 years, n (%)	311 (7%)
Sex, n (%)	
Missing	2,667 (58%)
Female	966 (49% of available records)
Male	972 (41% of available records)
Indication for monitoring, n (%)	
AF management	374 (8%)
Cryptogenic stroke	845 (18%)
Palpitations	501 (11%)
Post-AF ablation	184 (4%)
Suspected AF	850 (19%)
Syncope	1,593 (35%)

AF: atrial fibrillation.

*Values are shown as median [25^th^ percentile, 75^th^ percentile].

**Table 2:** Episode Transmission and Clinician View Times

Episode Type	Time from Episode to Merlin.net™ Availability	Time from Merlin.net™ Availability to Clinician View
Device initiated*	18.5 [11.2, 36.5] hours	0.8 [0.3, 2.5] days
Patient initiated*	2.9 [2.1, 3.8] minutes	0.9 [0.4, 3.1] days

*Values are shown as median [25^th^ percentile, 75^th^ percentile].

**Table 3:** Transmission Time by Episode Type

Episode Type	Number of Patients with Episode	Number of Episodes	Time from Detection to Merlin.net™ Availability*
Symptom	3,310	30,214	2.9 [2.1, 3.8] minutes
Bradycardia	386	81,345	17.4 [7.6, 28.1] hours
Pause	821	86,230	18.1 [9.0, 36.4] hours
Tachycardia	781	68,840	17.9 [11.3, 38.5] hours
Atrial fibrillation	1,206	95,850	19.2 [12.6, 38.0] hours

*Values are shown as median [25^th^ percentile, 75^th^ percentile].
